# Weight Gain during and after Pregnancy in Women with Gestational Diabetes Mellitus―A Preliminary Study

**DOI:** 10.3390/ijerph191911959

**Published:** 2022-09-22

**Authors:** Dorota Ćwiek, Anna Lubkowska, Małgorzata Zimny, Katarzyna Szymoniak, Olimpia Sipak-Szmigiel

**Affiliations:** 1 Department of Obstetrics and Pathology of Pregnancy, Pomeranian Medical University in Szczecin, 70-204 Szczecin, Poland; 2 Department of Functional Diagnostics and Physical Medicine, Pomeranian Medical University in Szczecin, 70-204 Szczecin, Poland

**Keywords:** pregnancy, body weight, gestational diabetes, BMI

## Abstract

Appropriate gestational weight gain (GWG) favors fewer complications related to pregnancy, delivery, puerperium, and the condition of the fetus and newborn baby. The aim of this study was to evaluate weight gain in women during and after pregnancy, including both women with and without gestational diabetes mellitus (GDM). Materials and methods: The study involved 42 singleton pregnant women diagnosed with GDM between the 24th and 28th week of pregnancy. The control group consisted of 28 nondiabetic women with a singleton pregnancy. The pre-pregnancy BMI, intra-pregnancy weight gain, and postpartum body weight were assessed in the participants. Results: There were no statistically significant differences in the values of intra-pregnancy weight gain. Only diabetic women who were also overweight or obese had a significantly higher percentage of weight gain during pregnancy. The analysis of the percentage of weight gain during the entire pregnancy showed differences only in the group of women with pre-pregnancy BMI over 30. Conclusions: There were no significant differences in total pregnancy or mid-pregnancy weight gain between women with and without GDM. Most of the women had too high or too low total-pregnancy and mid-pregnancy weight gain. Therefore it is necessary to control GWG and educate pregnant women about it.

## 1. Introduction

Pre-pregnancy weight and weight gain during pregnancy are important factors influencing maternal and child health. Obesity is increasingly recognized as a significant risk factor for miscarriage, preterm delivery, metabolic disorders complicating pregnancy, and higher rates of abnormal delivery, cesarean section, stillbirth, and neonatal death. Malnutrition may also contribute to lower neonatal weight, placental anomalies and related complications, higher rates of operative deliveries, including cesarean section, and higher fetal and neonatal mortality [1,2].

Normal GWG for women with a pre-pregnancy BMI below 18.5 is 0.51 kg per week in the second and third trimesters, and a total of 12.5–18 kg throughout pregnancy. For women with a pre-pregnancy BMI of 18.5–24.9, weight gain should be 0.42 kg per week in the second and third trimesters, and 11.5–16.0 kg throughout pregnancy. Women with a pre-pregnancy BMI of 25.0–29.9 should have a weekly weight gain of 0.28 kg in the second and third trimesters, and 7–11.5 kg throughout pregnancy. Women with grade I obesity (BMI of 30.0–34.9) should gain 4.5–11.0 kg (0.22 kg per week), with grade II obesity (BMI of 35.0–39.9) 0–4 kg, and with grade III obesity (BMI over 40.0) up to −4 kg throughout pregnancy [1].

Many authors have shown that GWG is a predictor of postpartum weight gain and may prevent obesity in middle-aged and elderly women [3]. Insufficient GWG was associated with decreased postpartum weight gain and a lower BMI, while excessive GWG was related to increased postpartum weight gain and a higher BMI [4].

Obesity in pregnant women is a big problem. In 2009, The Institute of Medicine (IOM, Washington, DC, USA) revised the guidelines for weight gain in pregnancy, including those for obese women. It recommended lower weight gain in obese women because higher GWG is positively associated with obstetric failure and higher neonatal morbidity and mortality [5]. Elevated pre-pregnancy BMI and excessive weight gain during pregnancy also correlate positively with the need for cesarean section and the occurrence of neonatal birth weight > 4000 g [6,7]. Obese women often experience reduced weight gain during pregnancy. According to Beyerlein et al., excessively low weight gain in obese pregnant women is associated with a reduced risk of pregnancy complications, such as pre-eclampsia and unplanned cesarean section, but unfortunately the risk of preterm and small gestational age (SGA) births increases significantly in overweight mothers and those with class 1 or class 2 obesity [8]. 

Gestational diabetes mellitus (GDM) and excessive or insufficient weight gain during pregnancy are also serious medical problems. Yee et al. found that excessive total weight gain in women with GDM was linked to increased neonatal morbidity. In contrast, better maternal and neonatal perinatal outcomes were observed in women with modest weight loss in the period after the diagnosis of GDM, although there was an increased incidence of preterm and SGA births in this group of mothers [5]. Similar conclusions were also reached by Blomberg, who noted that in a population of Swedish obese women (BMI > 35), both with and without GDM, who lost weight during pregnancy, there was an increased likelihood of having SGA neonates, but a lower likelihood of cesarean section and delivery of large gestational age (LGA) neonates [9].

Correct weight gain ranges during pregnancy are a very important issue related to good maternal and neonatal outcomes in all women [10]. Preventive measures can be taken while women are still of childbearing age, through implementing educational programs. 

The aim of this study was to evaluate the range of maternal weight gain during pregnancy and after delivery.

## 2. Materials and Methods

The study involved 42 singleton pregnant women diagnosed with GDM between the 24th and 28th week of pregnancy. The inclusion criteria were singleton pregnancy and a diagnosis of GDM. The control group consisted of 28 nondiabetic women with a singleton pregnancy. All subjects delivered after the completed 37th week of gestation. Each woman was examined twice―between the 24th and the 36th week of gestation and after delivery.

A pre-pregnancy BMI was determined based on height measured on the day of examination and pre-pregnancy weight reported in the history. The women underwent pregnancy weight measurement with a medical scale certified for medical (professional) use, meeting the Non-Automatic Weighing Instruments (NAWI) Directive and the Class III IOM.

Based on their BMIs, the women were categorized according to the classification proposed by The World Health Organization (WHO) [11]. The research covered a fairly long gestational period. Furthermore, the exact duration of pregnancy at the time of the examination was established based on the date of the last menstruation, verified by ultrasound. Taking into account this duration of pregnancy, based on the guidelines of the Institute of Medicine (IOM, Washington, DC, USA), the appropriate weight gain was determined with regard to a pre-pregnancy BMI per weeks of gestation up to the time of the study. For pregnant women with a low pre-pregnancy BMI (below 18.5 kg/m^2^), weight gain in the first trimester should be 2.3 kg, and in the following trimesters 0.51 kg (0.44–0.58 kg) per week. Pregnant women with normal weight (a BMI of 18.5–24.9 kg/m^2^) should increase their body weight in the first trimester by 1.6 kg, and in the following trimesters by 0.42 kg (0.35–0.50 kg) per week. In overweight pregnant women (a BMI of 25.0–29.9 kg/m^2^), weight gain in the first trimester should be 0.9 kg, and in the following trimesters 0.28 kg (0.23–0.33 kg) per week. Pregnant women with obesity (a BMI over 30 kg/m^2^) should increase their body weight by 0.22 kg (0.17–0.27 kg) per week [1,12]. Information on the subjects’ postpartum weight was obtained from the documentation confirmed by interview.

The data obtained were subjected to mathematical and statistical analysis (correlation analysis, chi-square test for independence, Student’s t-test, Mann–Whitney U test, and ANOVA were used).

The research protocol was approved by the Bioethics Committee of the Pomeranian Medical University in Szczecin (no. KB-0012/75/2015 of 22 June 2015).

## 3. Results

Statistical analysis showed that the compared groups did not differ statistically in any of the analyzed variables. The mean age of patients with GDM was 32.3 years, and the mean age of those without GDM was 30.57 years. Most of the respondents were married (71.4% in the group with GDM and 67.8% in the control group), lived in the city (92.9% and 89.3%, respectively), had good financial status (61.9% and 71.4%, respectively), were employed (83.3% and 71.4%, respectively), and had higher education (88.1% and 67.9%, respectively). Most of the subjects were primiparas (50.0% and 64.3%, respectively), and the mean birth weight of a baby in the group with GDM was 3429.17 g, and in the group without GDM was 3375.36 g. Analysis of a pre-pregnancy BMI showed no significant difference between the groups (*p* > 0.2863). The mean BMI for the subjects with GDM was 25.15, and for those without GDM was 23.98. The largest number of respondents had a normal BMI (26 and 13 subjects, respectively). There were no significant differences between the groups in terms of the time when the women were tested. Up to the 25th week of pregnancy, 33.3% of women with GDM and 21.43% without GDM were examined. 33.3% of patients with GDM and 32.14% without this disease were included in the study between the 26th and the 30th week of gestation. The rest of the respondents were qualified between the 31st and 36th week of pregnancy. All of the women diagnosed with GDM had received diabetes education and were under the supervision of a diabetologist, and their blood glucose levels were within normal ranges. They were regulated by diet or diet and insulin. The data are presented in Table 1.

**Figure 1:**
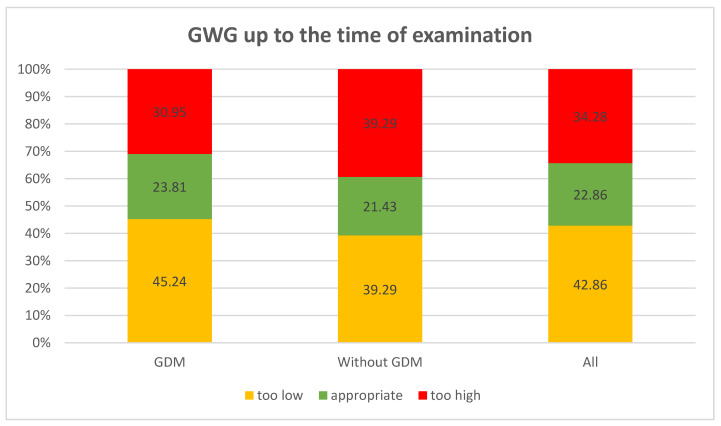
Gestational weight gain up to the time of examination.

**Figure 2:**
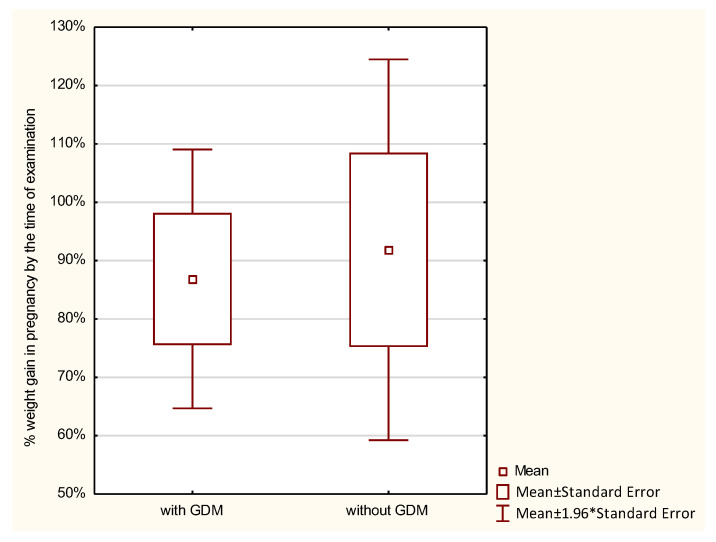
The percentage of GWG by the time of examination performed in pregnancy in relation to GDM.

**Figure 3:**
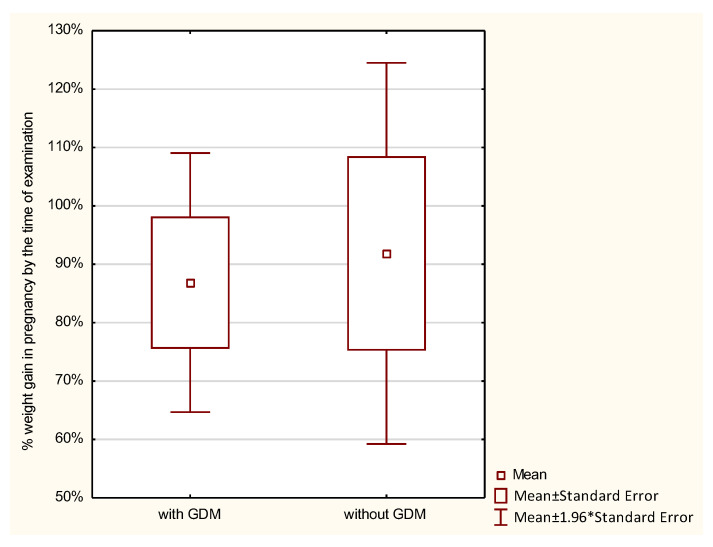
The percentage of GWG by the time of examination performed in pregnant women with a BMI < 18.5.

**Figure 4:**
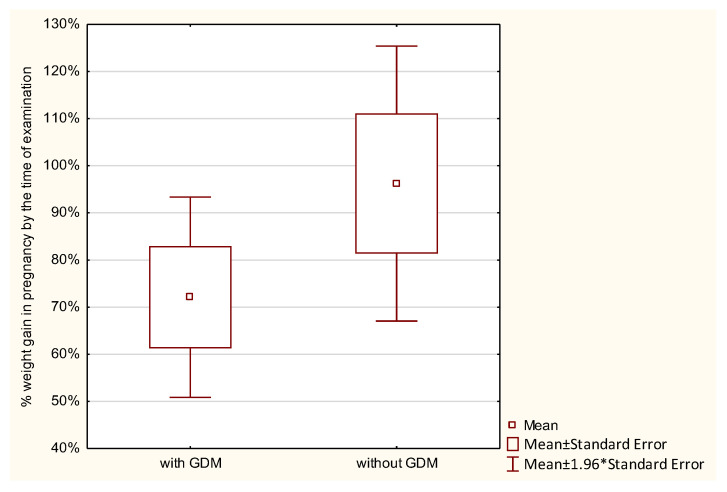
The percentage of GWG by the time of examination performed in pregnant women with a BMI of 18.5–24.9.

**Figure 5:**
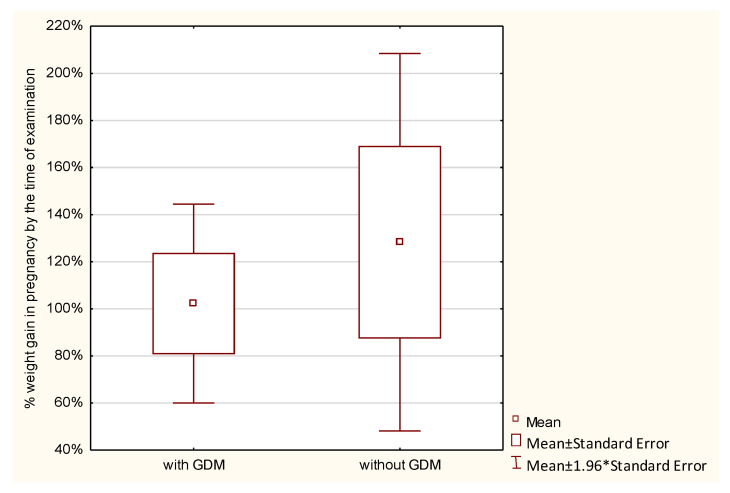
The percentage of GWG by the time of examination performed in pregnant women with a BMI of 25.0–29.9.

**Figure 6:**
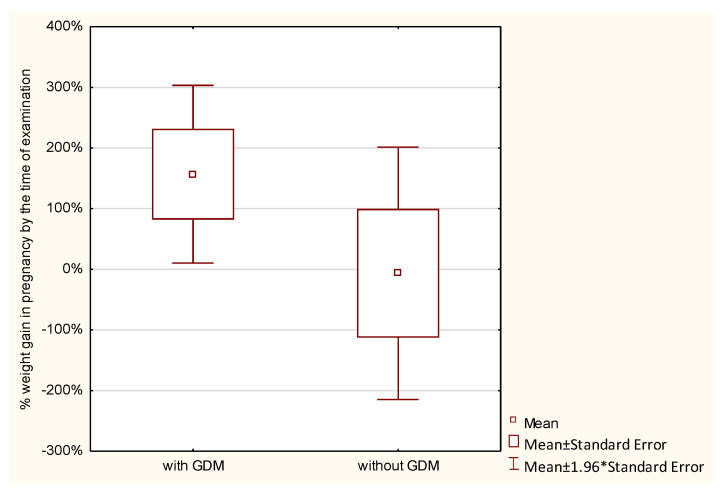
The percentage of GWG by the time of examination performed in pregnant women with a BMI > 30.0.

Weight gain during pregnancy was analyzed in the studied women. Normal weight gain was observed in 23.8%, too low in 45.24%, and too high in 30.95% of women with GDM, and in 21.43%, 39.29%, and 39.29% of those without GDM, respectively; the differences were not statistically significant. GWG up to the time of examination was analyzed with reference to the recommendations of the Institute of Medicine (IOM). No statistically significant differences were found between the groups in the percentage of GWG up to the time of examination (*p* > 0.7976). Average weight gain in women with GDM constituted 86.87%, and in those without diabetes 91.86% of the reference weight. Analysis of the percentage of weight gain by the time of examination in relation to a pre-pregnancy BMI demonstrated that the higher the BMI in the GDM group, the higher the percentage of weight gain. The lowest weight gain was observed in women with a BMI < 18.5 (52.16% of the reference weight), then in women with a normal BMI (72.08% of the reference weight), followed by those overweight (102.24% of the reference weight), and the highest in women with obesity (156.9% of the reference weight). In the control group, weight gain in underweight women was 94.77%, in women with normal weight 96.23%, and in overweight women 128.26% of the reference weight. In this group, obese women had a weight loss of −6.66% of the reference weight. However, these differences were not statistically significant (Table 2 and Figure 1, Figure 2, Figure 3, Figure 4, Figure 5 and Figure 6).

ANOVA demonstrated that when comparing the subjects by the percentage of weight gain up to the time of examination, significant differences were seen for the combined effects of diabetes and a pre-pregnancy BMI. None of these factors separately differentiated the subjects in terms of the percentage of weight gain. Women with diabetes who were also overweight or obese had a significantly higher percentage of weight gain during pregnancy (Table 3).

**Figure 7:**
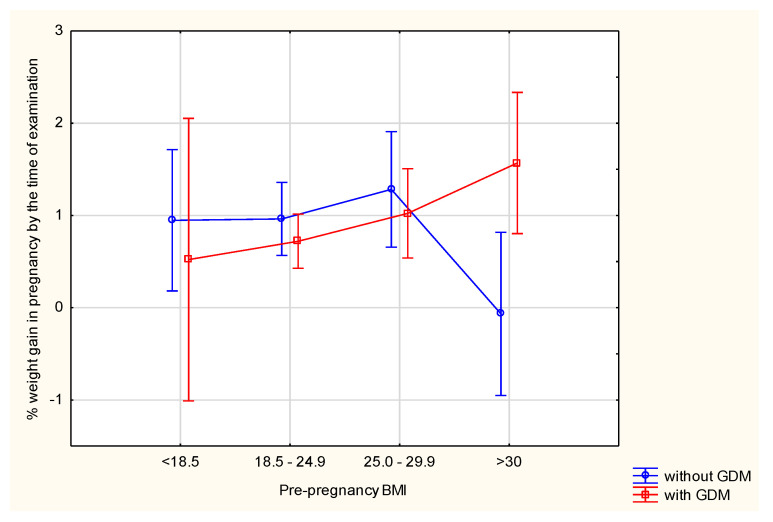
The effect of a pre-pregnancy BMI on the percentage of GWG by the time of examination.

We checked whether there were significant differences in the mean levels of these variables between the groups (GDM and non-GDM) and the variables studied in these groups (percentage of weight gain, pre-pregnancy BMI). Subsequently, we tested whether the change in percent weight gain was influenced by pre-pregnancy BMI in the groups of women with and without GDM. ANOVA analysis confirmed that there were differences in weight gain depending on the BMI in relation to diabetes. The least significant difference (LSD) test indicated between which groups these differences were statistically significant. Table 4 shows the categories which significantly differed in the percentage of weight gain (marked in red). Thus, it can be concluded that significant differences in weight gain by the time of examination occurred between the group (8) without GDM and with obesity and: the group (6) without GDM and with a BMI in the range of 18.5–24.9 (*p* < 0.037660); the group (7) without GDM and with normal weight (*p* < 0.015413); the group (3) with GDM and overweight (*p* < 0.034646); and the group (4) with GDM and obesity (*p* < 0.006877). Statistically significant differences were also observed between the group (4) with GDM and obesity and the group (2) with GDM and normal weight (*p* < 0.042900) (Table 4 and Figure 7).

Analysis of the percentage of weight gain during the entire pregnancy showed differences only in women with a pre-pregnancy BMI > 30. In this group, 141.48% weight gain was observed in subjects with GDM, while in those without GDM this was 62.12% (*p* < 0.005016). In women with GDM, total GWG is directly proportional to a pre-pregnancy BMI. There were no significant differences between the groups in terms of weight gain measured in kilograms in relation to a pre-pregnancy BMI (Table 5).

## 4. Discussion

Appropriate weight gain in women during pregnancy favors fewer complications related to pregnancy, delivery, puerperium, and the condition of the fetus and newborn baby. Obesity is increasingly recognized as one of the most important obstetric problems that increase the risk of maternal complications such as miscarriage, preterm delivery, metabolic disorders complicating pregnancy, as well as higher rates of abnormal birth, cesarean section, stillbirth, and neonatal death [1]. It has also been shown that excessive weight gain during pregnancy is considered a critical period because it affects a woman’s long-term weight control and increases the risk of obesity for the mother and the child, thus raising the risk of related diseases [2,13]. Malnutrition may also result in lower neonatal weight, placental anomalies and related complications, higher rates of operative deliveries including cesarean section, and higher fetal and neonatal mortality. A study by Nehring et al. demonstrated that a below-recommended weight gain during pregnancy was associated with lower postpartum weight during the six months following delivery [14].

In 1990, the Institute of Medicine (IOM, Washington, DC, USA) issued guidelines for weight gain during pregnancy. These guidelines recommend the optimal range of weight gain for women based on their pre-pregnancy BMI [15]. These guidelines were slightly revised in 2009 [12].

Analysis of the percentage of total weight gain during pregnancy showed that 35.71% of the subjects gained weight either below or within the recommended range, while 28.57% put on too much weight. The study by Stotland et al. found that 9.3% of the subjects had a target GWG above, 11.9% below, and 78.9% within the IOM guidelines [15]. Widen et al., on the other hand, demonstrated that 56% of the women had excessive, 27.0% normal, and 16.0% insufficient GWG [16]. In the study by Bodnar et al., too little weight was gained by 18.5% of the subjects, normal by 31.1%, and excessive by 50.4% [17]. Dalenius et al. obtained different results. They noted that 21.5% of the women gained less than the recommended weight based on the aforementioned guidelines, 30.6% gained within, and 48.0% gained more than the recommended range [18]. Karachaliou et al. found that 45% of the subjects in their study had excessive and 32% normal weight gain, while 23% put on too little weight [19]. In several studies conducted in the US, 30% to 40% of women achieved weight gain above or below the IOM recommended range [15].

Analyzing the percentage of total GWG with regard to a pre-pregnancy BMI, Kowal et al. showed that in the group of women with a normal BMI, 41.4% of the subjects gained too much weight, 34.5% put on weight within the normal range, and 24.1% gained too little weight. As for overweight women, 5.7% of the subjects gained too little weight in pregnancy, 26.8% put on weight within the normal range, and 67.6% put on too much weight [20]. In our study, 40.48% of the respondents with a normal BMI had too low GWG, 35.71% had GWG compliant with the IOM recommendations, and 23.81% had too high GWG. Among overweight women, 18.75% of the respondents had too little, 43.75% normal, and 37.5% too high GWG. Dalenius et al. noted that overweight (58.8%) and obese (55.6%) women were significantly more likely to gain more weight than recommended compared to their underweight (26.2%) and normal weight (38.6%) counterparts [18]. In the study by Haugen et al., among overweight primiparas and multiparas, 74.1% and 68.1%, respectively, had a GWG higher than that recommended by the IOM, and among obese women it was 66.3% of primiparas and 56.1% of multiparas. In our study, we obtained slightly different results, namely 37.5% of overweight and 42.86% of obese women. In the same study by Haugen et al., 26.8% of primiparas and 30.5% of multiparas with a BMI < 18.5 before pregnancy had a GWG lower than that recommended by the IOM. In our study, it was 60.0% of women in this group [21].

The average weight gain during pregnancy for the entire group in our study was 12.45 kg. This result is comparable to that in the study by Chung et al., in which it was 11.8 kg [22]. Analysis of average weight gain with regard to the BMI category revealed that women with a low BMI had mean GWG of 14.72 kg, those with a normal BMI 13.21 kg, those overweight 11.79 kg, and obese women 8.36 kg. In their study, Li et al. showed that women with a too low BMI had GWG of 16.5 kg, those with normal weight 17.7 kg, overweight women 18.1 kg, and those with obesity 17.3 kg [23]. Slightly different results were reported by Kowal et al., who noticed that the mean GWG for women with a low BMI was 17.4 kg, those with a normal BMI 16.4 kg, with overweight 15.6 kg, and with obesity 12.2 kg [20]. According to Karachaliou et al., obese women had an average GWG of 10.9 kg, overweight women 13.4 kg, normal weight women 14.6 kg, and underweight women 15.3 kg [19]. In our study, 28.56% were obese subjects, whose weight gain during pregnancy averaged 8.36 kg (min. −2.0 kg, max. 30.0 kg). Yee et al., on the other hand, concluded that those with a higher BMI were more likely to lose weight during pregnancy [5].

In their study, Kowal et al. found that over 60% of obese women gained more weight than the recommended 5–9 kg, with an average weight gain of 12.2 kg [20]. Haugen et al. observed among 14.4% of obese primiparas and 20.8% of obese multiparas that weight gain was too little—an average of 0.4 kg and 0.2 kg, respectively [21]. In our study, obese women had an average GWG of 8.36 kg and accounted for over 40% of the respondents. Siega-Riz et al. noted that GWG in obese women was higher than the recommended range of 5–9 kg, and that GWG decreased with the degree of obesity, but was higher than that mentioned in the 2009 IOM guidelines [24]. Different conclusions were reached by Nohr et al., who found that the average weight gain during pregnancy was less than 5 kg and decreased with increasing BMI. Obese women lost an average of 2 kg during pregnancy [25].

In our study, respondents with GDM compared to those without this condition in all groups had a lower weight gain. The only exception was obese women with GDM, whose mean GWG was significantly higher (141.48%) (*p* < 0.005), while their counterparts without GDM had GWG of −62.12%. Similar conclusions were reached by Karachaliou et al., who found that women with GDM had lower total GWG. The mean total GWG was 12.0 kg for women with GDM, and 14.1 kg for those without GDM [19]. Our results were similar: women with GDM had average total GWG of 11.47 kg, and those without GDM the total was 13.91 kg.

Mid-pregnancy weight gain was also analyzed in order to determine its compliance with the IOM recommendations. In over 40% of cases, mid-pregnancy weight gain was too low, in 34% it was too high, and only in less than 23% it was normal.

The effect of demographic and obstetric variables on GWG has not been investigated, but Karachaliou’s research demonstrated that well-educated women, multiparas, and women with a higher BMI before pregnancy had lower total GWG [19]. A pre-pregnancy BMI was strongly correlated with the target weight gain in women [15]. GWG is also influenced by other aspects, such as behavioral, mental, and social factors (including social support) [5,25].

Proper weight gain during pregnancy is very important because women who put on weight more than that recommended by the IOM tend to weigh more after delivery than women whose weight gain fell within the recommended range [26]. What is more, women who put on weight more or less than that recommended by the IOM are more likely to experience many adverse effects [15], including pregnancy, delivery, and postpartum disorders, as well as adverse health outcomes for the child and the woman.

## 5. Conclusions

Most of the women had too high or too low total pregnancy and mid-pregnancy weight gain, therefore it is necessary to control GWG and educate pregnant women about it. Pregnancy and childbirth are events that affect both the health of the woman and the baby. Therefore, individualized care and education are needed before a planned pregnancy, during pregnancy, and after delivery to help women achieve healthy weight gain during pregnancy and healthy weight postpartum. Women should be supported and motivated to achieve these goals.

## 6. Limitations

Several limitations of this study should be mentioned. The first is the small size of the study sample. This is a preliminary study that will be continued. The second is the homogeneity of the group―the study only involved women of similar ethnicity.

## Figures and Tables

**Table 1:** Sociodemographic data.

Sociodemographic and Obstetric Data		Whole Group n = 70 (%)	With GDM	Without GDM	*p*
			n = 42 (%)	n = 28 (%)	
**Marital status**	married	49 (70.0)	30 (71.43)	19 (67.86)	0.7494
	single	21 (30.0)	12 (28.57)	9 (32.14)	
**Education**	vocational	3 (4.29)	1 (2.38)	2 (7.14)	0.1159
	secondary	11 (15.71)	4 (9.52)	7 (25.00)	
	third-level	56 (80.0)	37 (88.09)	19 (67.86)	
**Place of residence**	the country	6 (8.57)	3 (7.14)	3 (10.71)	0.6010
	city	64 (91.43)	39 (92.86)	25 (89.29)	
**Financial status**	average	8 (11.43)	4 (9.52)	4 (14.29)	0.3561
	good	46 (65.71)	26 (61.90)	20 (71.43)	
	very good	16 (22.86)	12 (28.57)	4 (14.29)	
**Employment status**	employed	55 (78.57)	35 (83.33)	20 (71.43)	0.3032
	unemployed	15 (21.43)	7 (16.67)	8 (28.57)	
**Number of births**	1	39 (55.71)	21 (50.00)	18 (64.29)	0.3372
	2	22 (31.43)	16 (38.09)	6 (21.43)	
	3 or more	9 (12.86)	5 (11.90)	4 (14.29)	
**Pre-pregnancy BMI**	<18.5	8 (11.43)	2 (4.76)	6 (21.43)	0.2863
	19.8–24.9	39 (55.71)	26 (61.9)	13 (46.43)	
	25.0–29.9	16 (22.86)	10 (23.81)	6 (21.43)	
	>30.0	7 (10.0)	4 (9.52)	3 (10.71)	
	mean	24.68	25.15	23.98	
**Time of pregnancy test**	up to 25 weeks	20 (28.57)	14 (33.33)	6 (21.43)	0.4521
	26–30 weeks	23 (32.85)	14 (33.33)	9 (32.14)	
	31–36 weeks	27 (38.57)	14 (33.33)	13 (46.29)	
**Sociodemographic and obstetrical data**			**mean ± SD**	**mean ± SD**	*p*
**Age**	mean ± SD	31.61 ± 4.72	32.31 ± 4.51	30.57 ± 4.90	0.1319
**Baby’s birth weight**	mean ± SD	3413.43 ± 444.76	3429.17 ± 376.33	3375.36 ± 537.27	0.6235

n—number of respondents, SD—standard deviation, *p*—statistical significance, GWG—gestational weight gain, GDM—gestational diabetes mellitus, BMI—body mass index.

**Table 2:** GWG up to the time of examination.

GWG up to the Time of Examination		Whole Group n = 70		With GDM	Without GDM	*p*
				n = 42 (%)	n = 28 (%)	
**GWG**	too low		30 (42.86)	19 (45.24%)	11 (39.29%)	0.7707
	appropriate		16 (22.86)	10 (23.81%)	6 (21.43%)	
	too high		24 (34.28)	13 (30.95%)	11 (39.29%)	
**Percentage of GWG**	average		88.86%	86.87%	91.86%	0.7976
	min		−217.39%	−76.17%	−217.39%	
	max		371.43%	371.43%	275.36%	
	SD		0.7899	0.7334	0.8810	
**GWG by the time of examination and a pre-pregnancy BMI**	<18.5		average	52.16%	94.77%	0.510248
			min	52.16%	25.84%	
			max	52.16%	136.09%	
			SD	0.00	51.14%	
	18.5–24.9		average	72.08%	96.23%	0.194418
			min	−76.17%	7.76%	
			max	144.23%	173.01%	
			SD	0.563626	0.576515	
	25.0–29.9		average	102.24%	156.90%	0.543924
			min	−22.22%	32.43%	
			max	245.61%	371.43%	
			SD	0.681290	1.495549	
	>30.0		average	128.26%	−6.66%	0.248956
			min	13.89%	−217.39%	
			max	275.36%	121.95%	
			SD	1.001201	1.839757	

n—number of respondents, SD—standard deviation, min—minimum, max—maximum, *p*—statistical significance, GWG—gestational weight gain, GDM—gestational diabetes mellitus, BMI—body mass index.

**Table 3:** The percentage of GWG in relation to a pre-pregnancy BMI.

Variable	The Percentage of GWG in Relation to a Pre-Pregnancy BMI		
	F	*p*	Power Observed (Alpha = 0.05)
Diabetes	0.38758	0.535860	0.094032
Pre-pregnancy BMI	0.75451	0.523928	0.202454
diabetes * pre-pregnancy BMI	3.12724	0.031993	0.700893 *

* Significant differences (*p* < 0.05); Fisher test; BMI—body mass index.

**Table 4:** The least significant difference (LSD) test for individual study groups with regard to a BMI and GDM.

		The LSD Test for Individual Study Groups with Regard to a BMI and the Presence of GDM								
			With GDM				Without GDM			
			1	2	3	4	5	6	7	8
		Pre-Pregnancy BMI	below 18.5	18.5–24.9	25.0–29.9	30 or More	below 18.5	18.5–24.9	25.0–29.9	30 or More
**With GDM**	1	below 18.5		0.799243	0.535252	0.225873	0.620533	0.579442	0.361139	0.508459
	2	18.5–24.9			0.291538	0.042900 *	0.582328	0.331342	0.109148	0.096181
	3	25.0–29.9				0.232262	0.869509	0.848123	0.512967	0.034646 *
	4	30 or more					0.255652	0.164189	0.564527	0.006877 *
**Without GDM**	5	below 18.5						0.973059	0.500545	0.087902
	6	18.5–24.9							0.389839	0.037660 *
	7	25.0–29.9								0.015413 *
	8	30 or more								

GDM—gestational diabetes mellitus, BMI—body mass index, * significant differences (*p* < 0.05).

**Table 5:** Total GWG [kg] and the percentage of total GWG in relation to a pre-pregnancy BMI.

Percentage of Total GWG		Whole Group n = 70 (%)	With GDM	Without GDM	*p* Student’s *t*-Test
			n = 42 (%)	n = 28 (%)	
**Whole group**	average	37.71%	41.83%	31.53%	0.4111
	min		−74%	−100.0%	
	max		190.91%	126.1%	
	SD		0.7025	0.6771	
**Pre-pregnancy BMI < 18.5**	average	17.28%	−12.0%	24.6%	0.851630
	min		−12.0%	−52.0%	
	max		−12.0%	54.4%	
	SD		0.00	0.4361	
**Pre-pregnancy BMI** **18.5–24.9**	average	28.98%	24.57%	36.93%	0.546313
	min		−74.0%	−56.5%	
	max		75.0%	62.5%	
	SD		0.6626	0.5672	
**Pre-pregnancy BMI** **25.0–29.9**	average	59.78%	53.97%	69.46%	0.624101
	min		−42.9%	−57.14%	
	max		79.9%	126.1%	
	SD		0.5697	0.7060	
**Pre-pregnancy BMI > 30.0**	average	54.22%	141.48%	−62.12%	0.005016 *
	min		100.0%	−100.0%	
	max		190.91%	13.64%	
	SD		0.4833	0.6561	
**Total GWG [kg]**		**Whole Group** **n = 70**	**With GDM**	**Without GDM**	
			**n = 42 (%)**	**n = 28 (%)**	
**Whole group**	too low	25 (35.71)	16 (38.09)	9 (32.14)	
	appropriate	25 (35.71)	17 (40.48)	8 (28.57)	
	too high	20 (28.57)	9 (21.43)	11 (39.29)	
**Pre-pregnancy BMI** **< 18.5**	too low	3 (60.0)	1 (100.0)	2 (50.0)	
	appropriate	1 (20.0)	0 (0.0)	1 (25.0)	
	too high	1 (20.0)	0 (0.0)	1 (25.0)	
**Pre-pregnancy BMI** **18.5–24.9**	too low	17 (40.48)	13 (48.15)	4 (26.67)	
	appropriate	15 (35.71)	10 (37.01)	5 (33.33)	
	too high	10 (23.81)	4 (14.81)	6 (40.0)	
**Pre-pregnancy BMI** **25.0–29.9**	too low	3 (18.75)	2 (20.0)	1 (16.67)	
	appropriate	7 (43.75)	5 (50.0)	2 (33.33)	
	too high	6 (37.5)	3 (30.0)	3 (50.0)	
**Pre-pregnancy BMI** **> 30.0**	too low	2 (28.56)	0 (0.0)	2 (66.67)	
	appropriate	2 (28.56)	2 (50.0)	0 (0.0)	
	too high	3 (42.86)	2 (50.0)	1 (33.33)	
***p*–** $\chi^{2}$ **test**		0.2511	0.2742	0.3977	
**Total GWG [kg]**		**Whole Group** **n = 70**	**With GDM**	**Without GDM**	***p* Student’s *t*-Test**
			**n = 42 (%)**	**n = 28 (%)**	
**Whole group**	average	12.45	11.47	13.91	0.1541
	min		3	−2	
	max		30	27.8	
	SD		6.0744	8.0979	
**Pre-pregnancy BMI** **< 18.5**	average	14.72	11.0	15.64	0.682741
	min		11.0	6.0	
	max		11.0	27.8	
	SD		0.00	9.2266	
**Pre-pregnancy BMI** **18.5–24.9**	average	13.12	11.86	15.37	0.074679
	min		3.0	5.0	
	max		28.0	26.0	
	SD		5.715	6.3821	
**Pre-pregnancy BMI** **25.0–29.9**	average	11.79	10.27	14.33	0.258597
	min		4.0	3.0	
	max		20.0	26.0	
	SD		4.3939	9.5009	
**Pre-pregnancy BMI** ** > 30.0**	average	8.36	12.0	3.5	0.353876
	min		3.0	−2.0	
	max		30.0	12.5	
	SD		12.5167	7.8581	

n—number of respondents, SD—standard deviation, min—minimum, max—maximum, * significant differences (*p* < 0.05), GWG—gestational weight gain, GDM—gestational diabetes mellitus, BMI—body mass index.
